# Evaluation of Nano‐Encapsulation of Sweet Lime Peel Extract Through Its Application on Irradiated Chicken Meat Patties

**DOI:** 10.1002/fsn3.70124

**Published:** 2025-03-26

**Authors:** Hadia Hanif, Muhammad Sajid Arshad, Waseem Khalid, Felix Kwashie Madilo, Muhammad Zubair Khalid, Abu Talah, Ayesha Siddiqa, Ayman Luqman, Sulaiman Ali Alharbi, Hossam M. Aljawdah

**Affiliations:** ^1^ Department of Food Science, Faculty of Life Sciences Government College University Faisalabad Pakistan; ^2^ Department of Agriculture and Food Systems The University of Melbourne Melbourne Australia; ^3^ University Institute of Food Science and Technology The University of Lahore Lahore Pakistan; ^4^ Department of Food Science and Technology Ho Technical Universityn Ho Ghana; ^5^ Institute of Food Science and Technology, Faculty of Natural and Applied Sciences Khawaja Fareed University of Engineering and Information Technology Rahim Yar Khan Pakistan; ^6^ Department of Botany and Microbiology College of Science King Saud University Riyadh Saudi Arabia; ^7^ Department of Zoology College of Science, King Saud University Riyadh Saudi Arabia

**Keywords:** chicken patties, gamma irradiation, nano‐encapsulation, physicochemical parameters, sweet lime peel powder

## Abstract

Study was aimed to investigate the combined effects of gamma irradiation and nano‐encapsulation of extracts (2 kGy + 3% SPP and 4 kGy + 3% SPP) prepared from sweet lime peel powder to evaluate the antioxidant properties of irradiated chicken meat patties. The impact of various treatments on irradiated chicken patties was evaluated on physicochemical properties, storage stability analysis, peroxide value (POV), total volatile basic nitrogen (TVBN) and thiobarbituric acid reactive substances (TBARS) by varying storage intervals (0, 7, and 14 days) at freezing temperature (−5°C). The results showed that the gamma irradiated samples (4 kGy) led to a substantial decrease in the microbiological load in SPP added treated samples (T_5_: 4 kGy + 3%SPP>T4:2 kGy 3% SPP>T_2_: 4 kGy) as compared to control. Other parameters including heme pigment, hunter's color value, POV, TBARS, and TVBN also varied among various treatments and storage intervals significantly (*p* < 0.05). The highest POV and TBARS with irradiation (4 kGy) observed in T_1_ was 0.46 ± 0.04 meq peroxide/kg and 0.68 ± 0.12 MDA/kg, respectively, at 14th day of storage. While the highest POV (0.55 ± 0.09 meq peroxide/kg) was observed in T_4_ treatment samples with 4 kGy + 3% SPP. Total phenolic content and antioxidant potential (DPPH) were also observed to be higher in treated samples (T_5_ 59.41% ± 0.03%; T_4_ 63.7 ± 0.05bmg/g GAE respectively) as compared to control groups during storage. Conclusively, study results confirmed that the sweet lime peel powder extracts encapsulation combined with gamma irradiation resulted in improved physiochemical and antioxidant characteristics along with microbial quality of chicken meat patties during storage at freezing temperature.

## Graphical abstract

Irradiated chicken patties were evaluated for the physicochemical properties by applying various treatments (irradiation) and encapsulation of sweet lime peel powder extract (SPPE) to the formulation by varying storage intervals at freezing temperature. Physiochemical analysis including phenolic and antioxidant assays, heme pigments, storage stability, and sensory evaluation of irradiated and encapsulated patties showed a significant positive impact of treatments on evaluated parameters. The SPPE supplemented chicken meat patties combined with gamma irradiation resulted in a higher impact on the physicochemical and antioxidant characteristics than the irradiation alone.

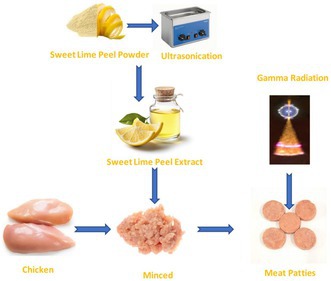

## 1 Introduction

Rising consumer demand for meat products owing to their widely recognized nutritional benefits led to substantial growth in the meat industry. Consumers are increasingly drawn to chicken meat due to its high protein and lipid value, making this meat more appealing in terms of its nutritional composition. Preserving meat poses a significant challenge due to its vulnerability to microbial contamination from various sources. The food industry widely recognizes irradiation as a highly effective nonthermal decontamination technique as a preservation measure. It has been recorded as a safe and effective method for extending the duration that fresh beef and its products can be stored without spoiling (Panseri et al. 2022).

Encapsulation is an important technique to protect bioactive compounds from degradation. Encapsulation can be achieved by many techniques, such as microencapsulation and nano‐encapsulation. Due to the smaller size of the capsules, nano‐encapsulation is preferred over microencapsulation. Capsules at the nano‐scale exhibit more bioavailability and are more efficient in controlling their release as compared to larger capsules. Encapsulating bioactive compounds with nanocapsules creates a protective barrier that shields them. Various delivery methods are used to encapsulate bioactive compounds with a nano‐carrier that is resistant to enzymatic degradation, particularly in the gastrointestinal tract. Nano‐carriers such as chitosan, alginate, and zein are commonly employed, and different methods, including association colloids, nanoemulsions, nanoparticles, nanolaminates, and nanofibers/nanotubes are utilized (Noore et al. 2021).

Sweet lime has nutritional and medicinal attributes, and its peel is also abundant in pectin, known for its efficacy in reducing both blood sugar and blood cholesterol levels, making it a noteworthy contributor to potential health benefits. Sweet lime peel powder is widely recognized as a significant source of antioxidants, making it a valuable addition to numerous food products for its beneficial health characteristics (Kumari et al. 2020). Extract of sweet lime powder was reported to have good antioxidant activities and antimicrobial properties owing to bioactive compounds including phenolics, α‐terpineol, α‐pinene, β‐pinene, d‐limonene, linallol, bergamol, etc. (Thiruvalluvan et al. 2024; Arafat et al. 2020). Due to its characteristics, it can be utilized as an ingredient in functional food and for the development of food bioactive packaging materials.

Irradiation application in food processing is known for its effectiveness for product stability and quality characteristics. Food products subjected to gamma irradiation undergo exposure to gamma rays, a physical method employed for sterilization or decontamination purposes. The Food and Drug Administration (FDA) has approved the use of ionizing radiation at a maximum intensity of 4.5 kGy to eliminate foodborne bacteria and increase the lifespan of refrigerated meat products (Sahoo et al. 2023). Food irradiation involves the use of controlled amounts of either nonionizing or ionizing radiation to process and treat various foods. Being a nonthermal physical method, food irradiation exhibits a beneficial impact on harmful microorganisms, spoilage agents, and even viruses. The nutritional composition of meat is extensively recognized, encompassing essential components such as high‐quality proteins, vitamins, trace minerals, and lipids (Islam et al. 2019). This method has no adverse effects on the sensory, physicochemical, or nutritional characteristics of the food products, and it may be applied to a wide variety of food items without diminishing their value (Bhatnagar et al. 2022). Irradiation is a preservation method that can effectively prolong the shelf life of meat by several days. Significant inactivation of *L. monocytogenes* , *S. typhimurium* , and *E. coli* in ready‐to‐cook chicken was achieved through the use of gamma radiation at various intensities (0, 1.5, 3, and 4.5 kGy) over the storage period. The chicken that was ready to consume maintained its sensory and textural qualities even after being stored for 15 days (Ibrahim 2013; Khalid et al. 2023).

The objective of this study was to evaluate the effects of incorporating nano‐encapsulated sweet lime peel powder extract on the quality and stability of chicken meat patties that have been irradiated. Chicken meat patties were subjected to varying levels of gamma irradiation alone, 2 and 4 kGy, and by the combined effect of sweet lime powder supplementation (3%) to assess their quality and durability during varied storage periods (0, 7, and 14 days) at freezing temperature (−5°C).

## 2 Materials and Methods

### 2.1 Procurement of Raw Material

The raw materials used in the study, including chicken and sweet lime, were procured from the local market in Faisalabad, Pakistan. Additionally, the chemicals and glassware required for the study were obtained from the labs of the Department of Food Science, Government College University Faisalabad, Pakistan.

### 2.2 Preparation of Sweet Lime Peel Powder

Initially, sweet limes were washed with tap water to remove dirt and impurities, and subsequently, their outer skin was carefully removed. The peels were then subjected to drying in a hot air oven at approximately 50°C. Using a grinder, the dried sweet lime peels were finely powdered. Then, the powder of sweet lime peel was carefully stored in an airtight container to safeguard it from potential moisture absorption from the surroundings.

### 2.3 Extraction by Ultrasonication

The extraction from sweet lime peel powder was carried out by the method described by Liu et al. (2014) by combining the powder in a beaker containing 70% ethanol at a 1:10 (solid‐to‐solvent ratio). Then ultrasonication was used to carry out the extraction, with a frequency of 20 kHz and an amplitude of 50% for 10 min at room temperature (25°C). The extract was obtained and filtered using Whatman No. 1 filter paper and subjected to reduced pressure at a rotary evaporator (Rotavac Valve Tec (EU), Germany) to get the concentrated extract.

### 2.4 Preparation of Emulsion and Spray Drying for Encapsulation

Sweet lime peel powder extract was first prepared and used here to make the emulsions. SPP‐based emulsions were prepared by using soy lecithin (1%) as the emulsifier (Samantha et al. 2015). The maltodextrin (MD) and gum arabic (GA) were homogenized in an equal ratio (1:1) to form a stable emulsion mixture (Ferrari et al. 2011). Subsequently, the emulsified samples were subjected to homogenization at 10,000 rpm for 10 min and spray dried (Spray Drier, TP‐S15) at 125°C inlet air temperature (IAT) with 5% wall material (WM) at 5 s needle speed to form spray‐dried nanocapsules (SDNs).

### 2.5 Prepration of Chicken Meat Patties

Chicken meat (Broiler) was purchased from Meat Mart, Faisalabad, and minced in a Commercial Meat Grinder Mincer (Westpoint Deluxe Meat Grinder WF‐1036) in the Food Grade Lab at GCUF. After mincing the meat, powdered sweet lime peel was incorporated into the mixture according to the treatment plan (Table 1) and then the mixture was shaped into patties. SPP was added at 3% in each patty made to a total weight of 100 g. Samples were irradiated by using gamma radiation Gamma Cell 220 (MDS Nordion, Ottawa, Canada) at the Nuclear Institute for Agriculture & Biology (NIAB), Faisalabad, Pakistan. Samples irradiated with and without SPP were subjected to two different radiation doses: 2 and 4 kGy (based on previous data reported highest effectiveness for chicken meat quality and stability) (Arshad et al. 2019). After the radiation treatment, the samples were aerobically packed in oxygen impermeable plastic films and subjected to storage in a freezer at −5°C. Subsequently, the samples were subjected to physicochemical analysis at 0, 7, and 14 days, respectively.

**TABLE 1:** Treatment plan for chicken meat patties prepared with sweet lime peel powder.

Treatments	Irradiation	Sweet lime peel powder
T_0_	—	—
T_1_	2 kGy	—
T_2_	4 kGy	—
T_3_	—	3% SPP
T_4_	2 kGy	3% SPP
T_5_	4 kGy	3% SPP

### 2.6 Microbial Analysis

#### 2.6.1 Total Viable Count

The various microbiological traits of chicken meat patties was examined by using the method of Harrigan and MacCane (1998). Sample 1 mL aliquots were aseptically transferred into sterile petri dishes with 10 mL of plate count agar (PCA) mixed well with melted, cooled aagr mixture. The medium and inoclums were well mixed and incubated at 37°C for 24 h. The total viable count in chickemn meat patty samples was determined by using the formula;

$$\left(S/S+D\right)\times \mathrm{PC}=\mathrm{TVC}$$

#### 2.6.2 Total Coliform Count

The amount of total coliform count (TC) was calculated by using the procedure of Helrich (1990) to estimate the coliform bacteria's like *E. coli* and *Enterobacter* in chicken patties during storage. Samples were prepared in enrichment broth buffered peptone water and homogenized. After preparing dilutions with sterile diluent (1:9 mL), 0.1 mL of each dilution was spreaded on agar plate and incubated at 37°C 24 h. The results were expressed as log CFU/g after calculating by using formula:

TC=No.of colonies×Dilution factor

### 2.7 Physiochemical Analysis of SPP Patties

#### 2.7.1 Heme Pigments

Krzywicki (1982) method was used for the determination of heme pigments, such as myoglobin (Mb), oxymyoglobin (MbO_2_), and metmyoglobin (MMb).

#### 2.7.2 Hunter's Color

The color of the chicken patties containing SPP was evaluated using a Hunter colorimeter. The color characteristics of chicken patties were evaluated by using Hunter's colorimeter using CIE Lab values indicating CIE L* (indicating lightness), a* (denotes red color), and CIE b* (indicating yellowness). Statistical assessment was carried out to calculate the average values of these color attributes.

### 2.8 Antioxidant Profile

#### 2.8.1 DPPH (2,2‐Diphenyl‐1‐Picrylhydrazyl) Scavenging Assay

The DPPH value of chicken patties was assessed using a modified version of the method described by Mimica‐Dukic et al. (2004). A homogenized chicken meat patties extract sample (0.2 mL) was combined with a freshly prepared solution of 0.0012 M (2,2‐diphenyl‐1‐picrylhydrazyl) and left to incubate at room temperature for 60 min. A spectrophotometer (U‐2900UV/VIS) was used to measure the absorption at 517 nm. The reduction in DPPH was determined as the value for each sample. The highest free RSA (Radical Scavenging Activity) of each specimen was displayed as a percentage reduction in DPPH and calculated by using the formula of DPPH absorbance at the start and after 60 min.

$$\begin{array}{cc} \text{DPPH inhibition}\%= & \left(\text{Initial absorbance}-\text{Final absorbance}\right)/\\ & \text{Initial absorbance}\times 100 \end{array}$$

#### 2.8.2 Total Phenolic Contents (TPC)

TPC values of the chicken patties containing SPP were assessed using the Folin–Ciocalteu method (Tezcan et al. 2009). A solution containing 10% Folin–Ciocalteu reagent was prepared, and 1 mL of this solution was added to 0.5 mL of the sample with the targeted concentration. The mixture was mixed and allowed to stand for 6 min, after which 2 mL of a 20% NaCO_3_ solution was added. After the reaction was allowed to proceed for 60 min at 30°C, the phenolic content was measured using a spectrophotometer (U‐2900UV/VIS) set to a wavelength of 760 nm. To determine the value of phenolic content, a calibration curve was created and adjusted, and the results were expressed as the equivalent of 1 g of Gallic acid equivalent (GAE) per gram of the sample.

### 2.9 Stability Test

#### 2.9.1 Total Volatile Basic Nitrogen (TVBN)

The assessment of total volatile basic nitrogen (TVBN) was conducted in accordance with the procedure outlined by Karim (2002). Samples were combined with trichloroacetic acid (TCA) in a 1:2 (weight/volume) ratio before being homogenized in a blender at speed 2. The mixtures obtained were thereafter subjected to centrifugation for a duration of 5 min at a speed of 1100 g and filtered by using Whatman No. 1 filter paper. 25 mL of the processed sample was then transferred to the Kjeldahl processing chamber, and 5 mL of 10% sodium hydroxide solution was mixed to get TVBN.

#### 2.9.2 Thiobarbituric Reactive Substances (TBARS)

To assess the degree of lipid oxidation, the TBARS (Thiobarbituric Acid Reactive Substances) method was utilized with certain adaptations, following the procedure described by Ahn et al. (1998). The TBARS values were quantified as milligrams (mg) of malondialdehyde per kilogram (kg) of meat, and the calculation was performed using the following equation:

$$\mathrm{mg}\;\text{malondialdehyde}\;\mathrm{per}\;\mathrm{kg}\;\text{meat}=\left(\text{Sample absorbance}-\text{blank}\right)\times \text{Total sample volume}/\left(0.000156\times 1000\right)$$

#### 2.9.3 Peroxide Value (POV)

The peroxide value (POV) of chicken patties was measured by using the method of Sallam et al. (2004). For the determination of the POV, 3 g of chicken patties containing SPP was measured and subjected to a hot water bath at 60°C for 3 min to melt its lipid contents. Further, 30 mL of acetic acid‐chloroform solution (3:2 v/v) was mixed and stirred for 5 min to break down the fats. The solid particles in the mixture were removed by filtration (Whatman No. 1) and then 0.5 mL of potassium iodide was added with starch solution. The POV was determined by the titration method, and POV was calculated by using the following formula:

$$\left\{\left(S\times N\right)/W\right\}\times 100$$

In the formula, *S* represents the volume of titration in milliliters, *N* stands for the normality of the sodium thiosulfate solution (*N* = 0.01) and *W* denotes the weight of the sample in grams.

### 2.10 Sensory Evaluation of Chicken Patties

To conduct the sensory evaluation of the chicken patties, a panel of 30 trained panelists was engaged. These experts were presented with the chicken patties and instructed to assign ratings on a scale ranging from 1 to 9. In this scale, a score of 9 signified an extremely favorable liking of the product, a score of 1 indicated that it was extremely disliked, and the values in between represented varying degrees of response, including neither exceptionally good nor bad. The trained panelists possessed the requisite knowledge to competently assess the product and were tasked with evaluating aspects such as its visual appearance, texture, aroma, flavor, and overall acceptability, as described by Meilgaard et al. (2007).

### 2.11 Statistical Analysis

The data collected for different parameters was subjected to statistical analysis using the software package Statistic 8.1. The significance levels (*p* ≤ 0.05) were obtained using analysis of variance (ANOVA) with a three‐factor factorial design in a completely randomized design (CRD) (Steel and Torrie 1980). The means were subsequently compared utilizing the least significant difference (LSD) method. Three replicates were used for all parameters, except for the sensory evaluation and Hunter's color assessment. For Hunter's color, a total of nine readings were collected randomly, while for sensory scores, a panel of 30 trained assessors was chosen.

## 3 Results and Discussion

### 3.1 Microbial Quality

#### 3.1.1 Total Viable Count and Coliform Count

The microbial analysis consisted of evaluating the quantities of aerobic bacteria and coliform count in both untreated and treated samples (Table 2), utilizing SPP and gamma irradiation. The control sample showed the highest TVC value (7.44 ± 0.036 log CFU/g) on the 14th day of storage, while the lowest TC content was (2.16 ± 0.01 log CFU/g) observed with a gamma irradiation dose of 2 kGy (Day 0). The results indicated that maximum decontamination was achieved in T_5_ when applying a dose of 4 kGy along with 3% SPP throughout all storage periods, as no colonies were detected in those samples (T_4_ and T_5_). On the 14th day of storage, the control sample exhibited a higher coliform count (4.21 ± 0.020 log CFU/g), while a lower coliform count (2.81 ± 0.032 log CFU/g) was also observed by applying gamma irradiation dose (2 kGy) on Day 0 of storage. These findings showed that thorough decontamination was attained using a gamma irradiation dose of 4 kGy + 3% SPP throughout the entire storage intervals of 0, 7, and 14 days.

**TABLE 2:** Total viable and coliform count in chicken meat patties treated with gamma irradiation and sweet lime peel powder during storage.

Parameter	Treatments	Storage period (days)		
		0	7	14
Total viable count (log CFU/g)	T_0_	5.41 ± 0.01^ca^	6.20 ± 0.02^ba^	7.44 ± 0.03^aa^
	T_1_	2.16 ± 0.01^cc^	3.18 ± 0.03^bc^	4.23 ± 0.03^ac^
	T_2_	ND	2.8 ± 0.1^bd^	3.6 ± 0.25^ad^
	T_3_	3.5 ± 0.1^cb^	4.07 ± 0.06^bb^	5.19 ± 0.01^ab^
	T_4_	ND	ND	3.36 ± 0.15^ae^
	T_5_	ND	ND	ND
Total coliform count (log CFU/g)	T_0_	3.71 ± 0.02^ca^	3.85 ± 0.04^ba^	4.21 ± 0.02^aa^
	T_1_	2.81 ± 0.03^cc^	2.92 ± 0.03^bc^	3.52 ± 0.03^ac^
	T_2_	ND	ND	3 ± 0.1^ad^
	T_3_	3.20 ± 0.02^cb^	3.53 ± 0.03^bb^	3.6 ± 0.02^ab^
	T_4_	ND	ND	2.86 ± 0.15^ae^
	T_5_	ND	ND	ND

*Note:* T_0_ (Control), T_1_ (2 kGy), T_2_ (4 kGy), T_3_ (3% SPP), T_4_ (2 kGy + 3% SPP), and T_5_ (4 kGy + 3% SPP). Means carrying different capital letters in a columns differed significantly among treatments, whereas means carrying different small letters in columns differed significantly among storage days.

Study findings are in line with the results of Arshad et al. (2019), who found that higher radiation doses and longer storage times led to a decrease in bacteria and coliforms. They also discussed how using both radiation and antioxidants together effectively removed these bacteria during storage. Similarly, research reported that radiation lowered the number of bacteria and extended the shelf life of meat products, which supports our own results (An et al. 2017). Hocaoğlu et al. (2012) found that gamma irradiation decreased coliforms in shrimp, but their count increased over time during storage. Chen et al. (2017) reported that the fried chicken exposed to gamma rays caused a significant reduction in the microbial count of samples.

### 3.2 Physiochemical Assay

#### 3.2.1 Heme Pigments

Current study findings regarding the Mb content in chicken meat patties exhibited a significant effect based on the applied treatments and on the duration of storage. Sample treatment T_4_ with 2 kGy of gamma irradiation along with 3% sweet lime peel powder (SPP) had a higher Mb content (34.53% ± 0.03%) on the 0 day of storage, while those treated with 2 kGy alone showed a minimum content (T_1_: 16.45% ± 0.05%) on the 14th storage day, as shown in Table 3. Notably, the addition of Sweet lime peel powder contributed to an increase in Mb content for both treated and untreated chicken meat patties.

**TABLE 3:** Haem pigment of chicken meat patties treated with gamma irradiation and sweet lime peel powder during storage.

Parameter	Treatments	Storage period (days)		
		0	7	14
Myoglobin (%)	T_0_	33.27 ± 0.27^ab^	26.41 ± 0.02^be^	17.58 ± 0.02^cc^
	T_1_	32.84 ± 0.03^ac^	25.62 ± 0.13^bf^	16.45 ± 0.05^cf^
	T_2_	31.96 ± 0.03^ae^	27.35 ± 0.10^bd^	16.93 ± 0.05^ce^
	T_3_	31.43 ± 0.02^af^	27.97 ± 0.05^bc^	17.11 ± 0.01^cd^
	T_4_	34.53 ± 0.03^aa^	28.55 ± 0.05^ba^	18.60 ± 0.05^ca^
	T_5_	32.22 ± 0.03^ad^	28.10 ± 0.01^bb^	18.20 ± 0.08^cb^
Oxymyoglobin (%)	T_0_	13.44 ± 0.05^cc^	16.54 ± 0.13^bc^	21.66 ± 0.10^ac^
	T_1_	14.35 ± 0.25^cb^	17.55 ± 0.10^bb^	22.74 ± 0.23^ab^
	T_2_	14.86 ± 0.05^ca^	17.97 ± 0.12^ba^	22.98 ± 0.12^aa^
	T_3_	11.21 ± 0.10^cf^	15.52 ± 0.11^bd^	20.55 ± 0.11^ad^
	T_4_	12.22 ± 0.11^ce^	15.43 ± 0.15^be^	20.31 ± 0.1^af^
	T_5_	12.43 ± 0.1^cd^	14.50 ± 0.03^bf^	19.45 ± 0.1^ae^
Metmyoglobin (%)	T_0_	41.23 ± 0.12^cc^	52.46 ± 0.08^bc^	62.85 ± 0.04^ac^
	T_1_	45.25 ± 0.1^cb^	54.73 ± 0.1^bb^	65.50 ± 0.1^ab^
	T_2_	46.10 ± 0.10^ca^	55.43 ± 0.03^ba^	66.02 ± 0.11^aa^
	T_3_	36.21 ± 0.1^cf^	48.63 ± 0.16^bd^	58.22 ± 0.21^af^
	T_4_	36.89 ± 0.10^ce^	47.23 ± 0.09^be^	60.44 ± 0.53^ae^
	T_5_	37.54 ± 0.1^cd^	47.99 ± 0.11^bf^	62.50 ± 0.1^ad^

*Note:* T_0_ (Control), T_1_ (2 kGy), T_2_ (4 kGy), T_3_ (3% SPP), T_4_ (2 kGy + 3% SPP), and T_5_ (4 kGy + 3% SPP). Means carrying different capital letters in a columns differed significantly among treatments, whereas means carrying different small letters in columns differed significantly among storage days.

Storage samples results showed that the sample treatment T_2_ with at 4 kGy on the 14th day of storage had a higher MbO_2_ value (22.98% ± 0.12%), whereas the lowest value (11.21% ± 0.10%) was observed in T_3_ samples treated with 3% SPP on the 0 day of storage. The results indicated that the levels of MbO_2_ in the untreated chicken samples T_0_ (control group) exhibited a gradual increase over time. In addition, the inclusion of SPP resulted in a reduction in MbO_2_ levels in both the treated and untreated chicken meat patties. A higher MMb value (66.02% ± 0.11%) was noted in T_2_ treated samples (4 kGy) on the 14th day of storage, whereas the lowest value (36.21% ± 0.1%) was identified in T_4_ samples treated with 3% SPP on the 0 day of storage. The observations showed that the MMb content in untreated chicken patty samples (control) exhibited an increase as time progressed. In both treated and untreated chicken meat samples, the MMb contents demonstrated a decrease upon the addition of sweet lime peel powder (3%).

Present study results showed that the Mb pigment levels decreased, while the levels of MbO_2_ and MMb pigment increased in response to radiation dose and storage duration. These findings are consistent with the research conducted by An et al. (2017), which reported that heme pigments in irradiated smoked duck meat generally increased during storage, except for Mb. Another prior study highlighted that the presence of antioxidants in meat samples played a moderate role in mitigating Mb deterioration during processing, contributing to favorable heme‐pigment values (Cunha et al. 2018). Study results are further supported by the research findings that revealed that Mb content decreased with an increase in radiation doses and increased with the addition of antioxidants (Arshad et al. 2019). Both MbO_2_ and MMb values exhibited an increase as the storage time progressed. Our findings align with the studies of Reddy et al. (2015) who concluded that Mb oxidized into MMb over time and with increasing dose levels, following an intermittent process involving MbO_2_.

#### 3.2.2 Hunter Color

For color changes, both irradiated and nonirradiated chicken meat patties were evaluated, and notable color variations were observed. Statistical findings concerning the L* value of chicken meat samples indicate significant impacts based on treatments and storage duration. Treated samples T_4_ (3% SPP) showed a higher L* value (52.44 ± 0.13) on the 14th day of storage, whereas the lowest value (48.11 ± 0.12) was observed in T_1_ samples treated with a 2 kGy irradiation dose on day 0 of storage (Table 4). In addition, the L* values of both treated and untreated chicken meat patties increased by the addition of sweet lime peel powder. The statistical analysis showed that the a* value of chicken meat patties was significantly influenced by the treatments and storage intervals. A higher a* value (13.80 ± 0.18) was observed in treated samples T_5_ (4 kGy + 3% SPP) on day 0 of storage, while the lowest value (9.51 ± 0.1) was observed in untreated T_0_ samples on the 14th storage day. The results suggest that the a* value of untreated chicken samples T_0_ declined gradually over time. In addition, the incorporation of SPP into chicken meat patties resulted in an increase in the a* values in both the treated and untreated chicken meat samples.

**TABLE 4:** Hunter's color of chicken meat patties treated with gamma irradiation and sweet lime peel powder during storage.

Parameter	Treatments	Storage period (days)		
		0	7	14
L*	T_0_	48.22 ± 0.11^cd^	48.7 ± 0.09^bd^	49.12 ± 0.05^ad^
	T_1_	48.11 ± 0.12^ce^	48.21 ± 0.01^bf^	48.47 ± 0.1^af^
	T_2_	48.43 ± 0.44^bc^	48.35 ± 0.1^ce^	48.55 ± 0.11^ae^
	T_3_	51.09 ± 0.1^ba^	49.83 ± 1.13^cc^	52.44 ± 0.13^aa^
	T_4_	49.19 ± 0.09^cb^	50.11 ± 0.02^bb^	51.47 ± 0.005^ac^
	T_5_	49.12 ± 0.01^cb^	50.44 ± 0.11^ba^	51.77 ± 0.11^ab^
a*	T_0_	10.20 ± 0.08^ae^	9.76 ± 0.1^bf^	9.51 ± 0.1^cf^
	T_1_	10.23 ± 0.09^ae^	9.90 ± 0.08^be^	9.60 ± 0.11^ce^
	T_2_	10.78 ± 0.09^ad^	10.07 ± 0.32^bd^	9.7 ± 0.25^cd^
	T_3_	13.13 ± 0.15^ac^	12.2 ± 0.1^bc^	11.43 ± 0.11^cc^
	T_4_	13.45 ± 0.1^ab^	12.97 ± 0.04^bb^	12.72 ± 0.02^cb^
	T_5_	13.80 ± 0.18^aa^	13.42 ± 0.13^ba^	13.02 ± 0.07^ca^
b*	T_0_	7.10 ± 0.19^af^	7.02 ± 0.03^be^	6.89 ± 0.09^cf^
	T_1_	7.25 ± 0.1^be^	7.22 ± 0.11^cd^	7.47 ± 0.1^ae^
	T_2_	7.44 ± 0.15^cd^	7.53 ± 0.12^bc^	7.65 ± 0.20^ad^
	T_3_	12.22 ± 0.11^ac^	12.12 ± 0.12^bb^	12.02 ± 0.08^cc^
	T_4_	12.61 ± 0.23^cb^	12.74 ± 0.1^aa^	12.66 ± 0.11^bb^
	T_5_	12.72 ± 0.2^ca^	12.75 ± 0.09^ba^	12.81 ± 0.1^aa^

*Note:* T_0_ (Control), T_1_ (2 kGy), T_2_ (4 kGy), T_3_ (3% SPP), T_4_ (2 kGy + 3% SPP), and T_5_ (4 kGy + 3% SPP). Means carrying different capital letters in a columns differed significantly among treatments, whereas means carrying different small letters in columns differed significantly among storage days.

The results of our study are consistent with the findings of Yaqoob et al. (2020), who observed that the L* (lightness), a*, and b* values increase when different amounts of papaya leaf extract and guava leaf extract are added. However, the L* value decreases as the storage duration increases. Similarly, Mehrzadeh and Roomiani (2021) corroborated that the L*, a*, and b* values exhibit an upward trend with increasing radiation dose and a downward trend with the passage of time. Li et al. (2017) observed minimal alterations in meat color throughout storage. Ayari et al. (2016) reported an increase in L* value with an increase in irradiation doses in ground beef, which parallels our current outcomes. Furthermore, our current findings also matched the earlier study conducted by Zhao et al. (2017) on chopped beef, indicating that irradiation doses induce color changes in meat.

### 3.3 Stability Parameters

#### 3.3.1 Thiobarbituric Reactive Substances (TBARS)

In current research study, under varied treatments and storage interval, there were significant variations observed in the TBARS value of chicken meat patties. A higher TBARS value (0.68 ± 0.12 MDA/kg) was noted in treated samples T_2_ (4 kGy) on the 14th day of storage while the lowest value (0.28 ± 0.08 MDA/kg) was observed in T_3_ on 0 day of storage as shown in Table 5.

**TABLE 5:** Thiobarbituric acid reactive substances (TBARS) and peroxide value (POV) value of chicken meat patties treated with gamma irradiation and sweet lime peel powder during storage.

Parameter	Treatments	Storage period (days)		
		0	7	14
TBARS (MDA/kg)	T_0_	0.33 ± 0.11^cd^	0.36 ± 0.06^bd^	0.39 ± 0.03^ae^
	T_1_	0.41 ± 0.12^cb^	0.56 ± 0.03^bb^	0.60 ± 0.05^ab^
	T_2_	0.49 ± 0.09^ca^	0.63 ± 0.06^ba^	0.68 ± 0.12^aa^
	T_3_	0.28 ± 0.08^ce^	0.40 ± 0.2^bd^	0.48 ± 0.08^ad^
	T_4_	0.32 ± 0.22^cd^	0.47 ± 0.07^bc^	0.51 ± 0.10^ac^
	T_5_	0.39 ± 0.11^cc^	0.54 ± 0.04^bb^	0.58 ± 0.13^ab^
POV (meq peroxide/kg)	T_0_	0.25 ± 0.02^cd^	0.27 ± 0.04^bd^	0.31 ± 0.1^ad^
	T_1_	0.31 ± 0.10^cb^	0.34 ± 0.07^bc^	0.37 ± 0.03^ac^
	T_2_	0.38 ± 0.1^ca^	0.45 ± 0.05^bb^	0.46 ± 0.04^aa^
	T_3_	0.19 ± 0.02^ce^	0.53 ± 0.07^aa^	0.29 ± 0.09^be^
	T_4_	0.29 ± 0.18^cc^	0.33 ± 0.07^bc^	0.35 ± 0.03^ac^
	T_5_	0.36 ± 0.06^ca^	0.55 ± 0.09^aa^	0.40 ± 0.01^bb^

*Note:* T_0_ (Control), T_1_ (2 kGy), T_2_ (4 kGy), T_3_ (3% SPP), T_4_ (2 kGy + 3% SPP), and T_5_ (4 kGy + 3% SPP). Means carrying different capital letters in a columns differed significantly among treatments, whereas means carrying different small letters in columns differed significantly among storage days.

The findings suggest that the TBARS value in untreated chicken samples T_0_ exhibited a gradual increase over time. Moreover, the TBARS readings in both the treated and untreated chicken flesh samples exhibited a decline upon the inclusion of SPP. The results of our investigation are consistent with the findings of Yang et al. (2014), who observed that Atlantic salmon fillets exposed to greater levels of irradiation (varying from 0.5 to 3 kGy) and held at 4°C showed a significant increase in TBARS value. A similar trend was observed by Ayari et al. (2016), who examined ground beef and found an increase in TBARS value over storage time, with the highest value at the 14 day of storage and the lowest on the 0th day. Our results also exhibit similarity to those of Yu et al. (2018), where they observed a rise in TBARS value with extended storage duration. Mehrzadeh and Roomiani (2021) showed that TBARS values increased with prolonged storage times but decreased with gamma irradiation and modified atmosphere packaging of white shrimps.

#### 3.3.2 Peroxide Value (POV)

The POV of chicken samples showed a substantial fluctuations in response to different treatments and storage periods in our study. The POV (0.46 ± 0.04 meq peroxide/kg) was observed to be higher in the T_2_ samples (4 kGy) on the 14th day of storage. In contrast, T_3_ samples untreated and supplemented with 3% SPP exhibited the lowest POV (0.19 ± 0.02 meq peroxide/kg) (Table 5). The findings indicated an increasing trend in POV for untreated chicken samples (T_0_) as time progressed. In addition, both chicken samples that were treated and untreated showed a reduction in POV upon the addition of SPP (3%) with the lowest observed in T_4_ (0.29 ± 0.18 meq peroxide/kg).

The results of the study conducted by Özalp Özen and Soyer (2018) have direct relevance to the present investigation, which involves the preservation of fish by employing various plant extracts. The study found that with the increased concentrations of plant extract, there was a decrease in the POV. However, the POV value increased as the storage period progressed (Özalp Özen and Soyer 2018). Our results also align with the study by Noor et al. (2022), where an increase in gamma irradiation corresponded to increased POV values, which in turn increased during storage.

In another study, Khalid et al. (2021) reported similar results with wheat germ and bran for both raw and cooked chicken patties during storage. In both types of patties, increasing concentration led to a reduction in POV, while storage time resulted in an increase. Du and Li (2008) further support these findings, as they observed a decrease in POVs in beef samples through the use of EO in the deep frying method.

#### 3.3.3 TVBN Assay

The outcomes concerning the TVBN measurement in chicken meat samples significantly varied upon varying treatments during the storage period. The highest TVBN value was observed in T_0_ (5.77 ± 0.11 mg Nitrogen/100 g) on 14 days of storage. While in treated samples, the lowest value (3.13 ± 0.05 mg Nitrogen/100 g) was observed in T_3_ (3% SPP) at 0 day of storage. The results demonstrated that the TVBN content in untreated chicken samples T_0_ progressively increased over time from 3.57 to 5.77 mg Nitrogen/100 g. However, upon irradiation in all treatments with irradiation and by the addition of SPP (3%), the increase of TVBN levels during storage was effectively inhibited till 14th day of storage (Table 6).

**TABLE 6:** Total volatile basic nitrogen (TVBN) of chicken meat patties treated with gamma irradiation and sweet lime peel powder during storage.

Parameter	Treatments	Storage period (days)		
		0	7	14
TVBN (mg Nitroge/100 g)	T_0_	3.57 ± 0.07^cd^	4.96 ± 0.12^ba^	5.77 ± 0.11^aa^
	T_1_	4.96 ± 0.02^ab^	3.87 ± 0.11^ce^	4.25 ± 0.05^bd^
	T_2_	5.08 ± 0.12^aa^	4.22 ± 0.11^cc^	4.55 ± 0.11^bc^
	T_3_	3.13 ± 0.05^ce^	4.86 ± 0.11^ab^	4.75 ± 0.10^bb^
	T_4_	3.99 ± 0.10^ac^	3.56 ± 0.24^cf^	3.86 ± 0.02^be^
	T_5_	4.95 ± 0.15^ab^	3.94 ± 0.05^bd^	4.33 ± 0.15^cf^

*Note:* T_0_ (Control), T_1_ (2 kGy), T_2_ (4 kGy), T_3_ (3% SPP), T_4_ (2 kGy + 3% SPP), and T_5_ (4 kGy + 3% SPP). Means carrying different capital letters in a columns differed significantly among treatments, whereas means carrying different small letters in columns differed significantly among storage days.

Our findings align with Dogruyol and Mol (2017), who observed that nonirradiated samples exhibited an increase in TVBN while the irradiated samples demonstrated a decrease in TVBN value throughout the storage period (40‐days). Another investigation by Yang et al. (2014) revealed an increase in TVBN as the storage duration extended, which corresponds with the results of our present study. This suggests that a dose of 0.5 kGy effectively restrained TVBN values from increasing and caused significant differences between irradiated and nonirradiated samples.

### 3.4 Antioxidant Potential

#### 3.4.1 DPPH (2,2‐Diphenyl‐1‐Picrylhydrazyl) Scavenging Assay

The DPPH value of chicken meat patties, which were subjected to gamma irradiation and treated with Sweet lime peel powder, exhibited significant changes in response to different storage periods. The treated samples T_3_ (3% SPP) had a higher DPPH value (63.2% ± 0.1%) on the first day of storage, whereas the T_2_ samples treated with 4 kGy had the lowest value (44.10% ± 0.1%) on the 14th day of storage (Table 7). The analysis of antioxidant potential showed that upon the addition of sweet lime peel powder (SPP) to chicken meat patties, an increase in the DPPH value was observed in both irradiated and supplemented samples as compared to untreated chicken samples T_0_ (control) over the storage period. The results align with the previous research conducted by Yaqoob et al. (2020), which showed a notable decrease in DPPH values over time in all experimental groups. The findings of our investigation are consistent with the findings of Fernandes et al. (2018), who evaluated the antioxidant capacity of spray‐dried guava leaf extracts. Research conducted by Sohaib et al. (2012) reported that the chicken nuggets enriched with antioxidants alpha lipoic acid and alpha tocopherol acetate consistently showed better DPPH inhibition potential during storage. Database reported that the extracts of sage and vitamin E extract can increase the antiradical power compared to the control treatment (Mielnik et al. 2003; Fasseas et al. 2008).

**TABLE 7:** 2,2‐diphenyl‐1‐picrylhydrazyl (DPPH) and total phenolic content (TPC) of chicken meat patties treated with gamma irradiation and sweet lime peel powder during storage.

Parameter	Treatments	Storage period (days)		
		0	7	14
DPPH (%)	T_0_	50.41 ± 0.47^ae^	49.02 ± 0.07^bf^	47.2 ± 0.1^cd^
	T_1_	54.51 ± 0.1^ad^	52.61 ± 0.2^be^	45.8 ± 1.11^ce^
	T_2_	57.90 ± 0.10^ac^	54.2 ± 0.1^bd^	44.10 ± 0.1^cf^
	T_3_	63.2 ± 0.1^aa^	61.01 ± 0.01^ba^	58.04 ± 0.20^ca^
	T_4_	59.41 ± 0.03^ab^	57.1 ± 0.04^bb^	54.01 ± 0.40^cb^
	T_5_	57.94 ± 0.05^ac^	55.1 ± 0.03^bc^	53.10 ± 0.11^cc^
TPC (mg/g GAE)	T_0_	56.20 ± 0.09^af^	52.7 ± 0.1^bf^	50.2 ± 0.25^cd^
	T_1_	65.09 ± 0.19^ac^	62.21 ± 0.11^bc^	49.3 ± 0.12^ce^
	T_2_	70.06 ± 0.78^ab^	66.10 ± 0.11^bb^	47.3 ± 0.1^cf^
	T_3_	75.10 ± 0.11^aa^	70.10 ± 0.05^ba^	61.33 ± 0.12^ca^
	T_4_	63.7 ± 0.05^ad^	61.23 ± 0.1^bd^	57.36 ± 0.18^cb^
	T_5_	60.02 ± 0.07^ae^	58.7 ± 0.25^be^	53.04 ± 0.15^cc^

*Note:* T_0_ (Control), T_1_ (2 kGy), T_2_ (4 kGy), T_3_ (3% SPP), T_4_ (2 kGy + 3% SPP), and T_5_ (4 kGy + 3% SPP). Means carrying different capital letters in a columns differed significantly among treatments, whereas means carrying different small letters in columns differed significantly among storage days.

#### 3.4.2 Total Phenolic Contents (TPC)

Study results revealed that the total phenolic content (TPC) in chicken meat patties showed variations in response to different treatments and storage durations. The treated sample T_3_ (3% SPP) exhibited a higher TPC of 75.10 ± 0.11 mg/g GAE on the 0 day of storage, whereas the lowest value (47.3 ± 0.1 mg/g GAE) was noted in T_2_ samples treated with a 4 kGy dose on the 14th day of storage (Table 7).

The outcomes demonstrated a reduction in TPC over time in untreated chicken samples (control). The presence of SPP led to an increase in TPC for both treated and untreated chicken meat samples. Our findings align with Arshad et al. (2019) discovery, which suggested that TPC increased with the addition of turmeric powder to chicken meat and decreased with increasing irradiation dose and storage duration. Similarly, Kim et al. (2013) observed similar results in meat patties treated with tomato powder, where TPC decreased over prolonged storage duration. Results are also consistent with Sadiq et al. (2023) findings, revealing that the highest TPC value occurred in chicken patties treated with 3% guava leaf extract on the 0 day of storage.

### 3.5 Sensory Evaluation of Chicken Patties

Evaluations of chicken meat patties were conducted in order to determine the mean scores for sensory. Results showed that the sensory attributes exhibited by irradiated and supplemented patties resulted in a decrease in sensory score as the gamma irradiation dosage increased, as well as over extended storage periods. On the 0 day of storage, the sensory scores were as follows: appearance ranged from 7.80 ± 0.10 to 6.61 ± 0.09, texture from 7.81 ± 0.10 to 6.81 ± 0.07, taste from 7.80 ± 0.10 to 6.70 ± 0.10, odor from 7.73 ± 0.20 to 7.16 ± 0.10, and overall acceptability from 7.61 ± 0.30 to 6.92 ± 0.11, as shown in Table 8.

**TABLE 8:** Sensory evaluation (appearance, texture, taste, odor, and overall acceptability) of chicken meat patties treated with gamma irradiation and sweet lime peel powder during storage.

Parameter	Treatments	Storage period (days)		
		0	7	14
Appearance	T_0_	7.80 ± 0.10^aa^	7.51 ± 0.20^ba^	6.92 ± 0.08^ca^
	T_1_	7.61 ± 0.20^ab^	7.32 ± 0.20^bb^	6.81 ± 0.10^cb^
	T_2_	7.02 ± 0.19^bc^	7.21 ± 0.30^ac^	6.62 ± 0.10^cc^
	T_3_	7.00 ± 0.05^ac^	6.90 ± 0.40^bd^	6.41 ± 0.20^cd^
	T_4_	6.81 ± 0.10^ad^	6.51 ± 0.10^be^	6.12 ± 0.20^ce^
	T_5_	6.61 ± 0.09^ae^	6.31 ± 0.10^bf^	5.96 ± 0.20^cf^
Texture	T_0_	7.81 ± 0.10^aa^	7.41 ± 0.07^ba^	6.81 ± 0.10^ca^
	T_1_	7.61 ± 0.20^ab^	7.22 ± 0.20^bb^	6.50 ± 0.10^cb^
	T_2_	7.41 ± 0.10^ac^	7.16 ± 0.30^bc^	6.40 ± 0.10^cc^
	T_3_	7.16 ± 0.35^ad^	6.91 ± 0.10^bd^	6.33 ± 0.11^cd^
	T_4_	6.91 ± 0.18^ae^	6.82 ± 0.11^be^	6.21 ± 0.20^ce^
	T_5_	6.81 ± 0.07^af^	6.72 ± 0.10^bf^	6.11 ± 0.10^cf^
Taste	T_0_	7.80 ± 0.10^aa^	7.40 ± 0.10^ba^	6.80 ± 0.10^ca^
	T_1_	7.53 ± 0.30^ab^	7.21 ± 0.20^bb^	6.61 ± 0.30^cb^
	T_2_	7.52 ± 0.20^ab^	6.81 ± 0.30^bc^	6.51 ± 0.10^cc^
	T_3_	7.07 ± 0.16^ac^	6.61 ± 0.20^bd^	6.52 ± 0.20^cc^
	T_4_	6.81 ± 0.10^ad^	6.51 ± 0.30^be^	6.31 ± 0.10^cd^
	T_5_	6.70 ± 0.10^ae^	6.41 ± 0.10^bf^	6.21 ± 0.20^ce^
Odor	T_0_	7.73 ± 0.20^aa^	7.21 ± 0.20^ba^	6.71 ± 0.20^ca^
	T_1_	7.61 ± 0.20^ab^	7.13 ± 0.30^bb^	6.51 ± 0.30^cb^
	T_2_	7.62 ± 0.20^ab^	6.81 ± 0.10^bc^	6.21 ± 0.40^cc^
	T_3_	7.52 ± 0.11^ac^	6.52 ± 0.30^bd^	6.13 ± 0.30^cd^
	T_4_	7.21 ± 0.10^ad^	6.21 ± 0.20^be^	5.95 ± 0.25^ce^
	T_5_	7.16 ± 0.10^ae^	6.20 ± 0.20^be^	5.80 ± 0.20^cf^
Overall acceptability	T_0_	7.61 ± 0.30^aa^	7.41 ± 0.30^ba^	6.82 ± 0.10^ca^
	T_1_	7.51 ± 0.10^bb^	7.31 ± 0.10^cb^	6.71 ± 0.20^ab^
	T_2_	7.30 ± 0.26^ad^	7.11 ± 0.20^bc^	6.41 ± 0.10^cc^
	T_3_	7.41 ± 0.10^ac^	6.92 ± 0.10^bd^	6.22 ± 0.10^cd^
	T_4_	7.06 ± 0.15^ae^	6.72 ± 0.20^be^	6.20 ± 0.26^cd^
	T_5_	6.92 ± 0.11^af^	6.52 ± 0.11^bf^	5.82 ± 0.35^ce^

*Note:* T_0_ (Control), T_1_ (2 kGy), T_2_ (4 kGy), T_3_ (3% SPP), T_4_ (2 kGy + 3% SPP), and T_5_ (4 kGy + 3% SPP). Means carrying different capital letters in a columns differed significantly among treatments, whereas means carrying different small letters in columns differed significantly among storage days.

As storage durations increased, the sensory scores pertaining to the various attributes exhibited significant reduction. This decrease in sensory scores during storage could be attributed to increased lipid oxidation levels induced by irradiation, resulting in decreased acceptability. Nevertheless, the overall acceptability remained within the acceptable range. Study findings are supported by Khalid et al. (2021), who evaluated the ostrich and chicken meat subjected to varying irradiation doses and by adding the kale leaf powder. In another study, Fallah‐Araghi et al. (2012) observed that both irradiated and nonirradiated products exhibited strong appearance and other quality characteristics on the first day, but nonirradiated grilled chicken resulted in lower sensory scores in irradiated samples after 15 days of storage. The findings of the present study are similar to Arshad et al. (2019), who reported that the storage period resulted in a decrease in the quality attributes of chicken meat, including appearance, texture, taste, flavor, and overall acceptability, as the storage time increased. In another study, Zhang et al. (2016) found that the sensory scores of e‐beam‐irradiated and vacuum‐packaged grass carp surimi decreased during storage.

## 4 Conclusions

Overall, the study findings indicate that the use of various treatments, including irradiation alone and in combination with sweet lime peel powder extract, improved the quality of chicken meat patties and also helps to control the physicochemical characteristics during storage. For further improvement in quality and shelf stability of the chicken patties during prolonged storage, a high dosage (4 kGy) with the addition of sweet lime peel powder (SPP 3%). Conclusively, chicken patties containing SP powder exhibited a greater antioxidant profile than all other treated samples. Conclusively, the irradiation and plant material extracts like SPP have significant potential to control microbial deterioration and adverse physicochemical changes in chicken meat patties during prolonged storage.

## Author Contributions

**Felix Kwashie Madilo:** investigation (equal), software (equal), visualization (equal), writing – original draft (equal). **Hadia Hanif:** conceptualization (equal), data curation (equal), investigation (equal), writing – original draft (equal). **Muhammad Zubair Khalid:** conceptualization (equal), methodology (equal), visualization (equal), writing – review and editing (equal). **Ayman Luqman:** funding acquisition (equal), project administration (equal), supervision (equal), visualization (equal). **Ayesha Siddiqa:** data curation (equal), resources (equal), validation (equal), writing – review and editing (equal). **Waseem Khalid:** formal analysis (equal), investigation (equal), supervision (equal), writing – review and editing (equal). **Sulaiman Ali Alharbi:** investigation (equal), software (equal), validation (equal), writing – review and editing (equal). **Hossam M. Aljawdah:** supervision (equal), validation (equal), visualization (equal), writing – original draft (equal). **Abu Talah:** data curation (equal), project administration (equal), supervision (equal), writing – original draft (equal). **Muhammad Sajid Arshad:** conceptualization (equal), formal analysis (equal), methodology (equal), supervision (equal).

## Conflicts of Interest

The authors declare no conflicts of interest.

## Data Availability

The authors confirm that data generated or analyzed supporting the findings of this study are available within the article, and any further supporting material will be provided on request from the corresponding author.
